# Application of a Combined Model with Autoregressive Integrated Moving Average (ARIMA) and Generalized Regression Neural Network (GRNN) in Forecasting Hepatitis Incidence in Heng County, China

**DOI:** 10.1371/journal.pone.0156768

**Published:** 2016-06-03

**Authors:** Wudi Wei, Junjun Jiang, Hao Liang, Lian Gao, Bingyu Liang, Jiegang Huang, Ning Zang, Yanyan Liao, Jun Yu, Jingzhen Lai, Fengxiang Qin, Jinming Su, Li Ye, Hui Chen

**Affiliations:** 1 Guangxi Key Laboratory of AIDS Prevention and Treatment & Guangxi Universities Key Laboratory of Prevention and Control of Highly Prevalent Disease, School of Public Health, Guangxi Medical University, Nanning 530021, Guangxi, China; 2 Centers for Disease Control and Prevention, Heng County 530300, Guangxi, China; 3 Guangxi Collaborative Innovation Center for Biomedicine, Guangxi Medical Research Center, Guangxi Medical University, Nanning 530021, Guangxi, China; 4 Geriatrics Digestion Department of Internal Medicine, The First Affiliated Hospital of Guangxi Medical University, Nanning 530021, Guangxi, China; Kaohsiung Chang Gung Memorial Hospital, TAIWAN

## Abstract

**Background:**

Hepatitis is a serious public health problem with increasing cases and property damage in Heng County. It is necessary to develop a model to predict the hepatitis epidemic that could be useful for preventing this disease.

**Methods:**

The autoregressive integrated moving average (ARIMA) model and the generalized regression neural network (GRNN) model were used to fit the incidence data from the Heng County CDC (Center for Disease Control and Prevention) from January 2005 to December 2012. Then, the ARIMA-GRNN hybrid model was developed. The incidence data from January 2013 to December 2013 were used to validate the models. Several parameters, including mean absolute error (MAE), root mean square error (RMSE), mean absolute percentage error (MAPE) and mean square error (MSE), were used to compare the performance among the three models.

**Results:**

The morbidity of hepatitis from Jan 2005 to Dec 2012 has seasonal variation and slightly rising trend. The ARIMA(0,1,2)(1,1,1)_12_ model was the most appropriate one with the residual test showing a white noise sequence. The smoothing factor of the basic GRNN model and the combined model was 1.8 and 0.07, respectively. The four parameters of the hybrid model were lower than those of the two single models in the validation. The parameters values of the GRNN model were the lowest in the fitting of the three models.

**Conclusions:**

The hybrid ARIMA-GRNN model showed better hepatitis incidence forecasting in Heng County than the single ARIMA model and the basic GRNN model. It is a potential decision-supportive tool for controlling hepatitis in Heng County.

## Introduction

In China, the Guangxi Zhuang Autonomous Region has a large burden of hepatocellular carcinoma, which has led to enormous property and health consequences [1]. The hepatocellular carcinoma epidemic of Heng County is particularly serious in Guangxi [2]. Hepatitis, especially due to hepatitis B virus (HBV) infection, is a strong risk factor for hepatocellular carcinoma [3, 4]. Controlling the incidence of hepatitis is one of the most important measures to reduce the epidemic of hepatocellular carcinoma. The annual morbidity due to hepatitis in Heng County is higher than the average level in Guangxi, and is ranked first in legal infectious disease of Heng County [5, 6]. It has become a major public health problem in the county as well as in Guangxi. Moreover, Heng County has been the key location of the Guangxi Beibu Gulf Economic Zone in recent years, which brings with it a large temporary floating population. This is a new potential threat contributing to increasing the incidence of hepatitis. Therefore, several interactional measures should be taken to control the epidemic. Disease surveillance is currently the principal measure used. However, monitoring data only reflect the current situation of the epidemic. The interaction measures based on monitoring data usually show some lag, so an accurate prediction of the hepatitis epidemic is essential to making the correct public health policy decisions in advance. Hence, it is very important to develop a high accurate forecasting model.

Currently, several mathematical models based on linear presumptions are employed to predict the incidence of infectious diseases [7, 8]. Among them, the ARIMA model is the most popular method [9–12]. However, epidemic data usually contain linear and non-linear information. The ARIMA model can only analyze the linear part of the incidence data [13, 14]. In order to overcome this inherent defect of the ARIMA model, an artificial neural network (ANN) model, with great capability for flexible non-linear fitting, was used to the complement the ARIMA model [15, 16]. Generally, it has been accepted that a hybrid model shows greater performance, and these models have been employed to analyze information from complicated series [17–19]. The GRNN model is a member of the ANN family with important characteristics of accelerated learning and greater capability for non-linear fitting [15]. This model also does well in forecasting the epidemic situation [20]. Several previous studies has shown that the combined ARIMA-GRNN model provides better incidence forecasting than the single ARIMA model [21–23], but there has been little research comparing the hybrid ARIMA-GRNN model with the basic GRNN model. Thus, it is unknown as to which model is the best among the three models. Thus, we conducted research to develop a single ARIMA model, a basic GRNN model and a hybrid ARIMA-GRNN model to predict the monthly morbidity of hepatitis. It is worth mentioning that we present a better method to develop the optimum GRNN model. The fitting and forecasting performance parameters of the combined model were compared with the single ARIMA model and the basic GRNN model so as to determine the best model. The model will be employed to provide reference information for hepatitis control and intervention. At the same time, it can be used to evaluate the effect of related interventions.

## Materials and Methods

### Materials Source

An ethical statement is not required for this study because these are secondary data for public access.

The monthly morbidity data for hepatitis in Heng County from January 2005 to December 2013 came from the Heng County CDC (Center for Disease Control and Prevention). The Heng County Statistics Bureau releases the population data. All hepatitis cases were primarily screened according to clinical symptoms and then confirmed by the assessment of antibody and pathogen levels. Subsequently, the data were collected by diagnostic case number according to the laboratory examination results.

All hepatitis cases must be reported within 12 hours to the Heng County CDC through an Internet-based disease-reporting system. It is assumed that the degree of compliance with disease notification over the study period was excellent due to compulsory reporting.

### Single ARIMA model construction

The ARIMA model is usually written in shorthand as ARIMA (p,d,q) (P,D,Q) _s_: p, the order of auto-regression; d, the degree of difference; q, the order of the moving average, P, the seasonal auto-regression lag; D, the degree of seasonal difference; Q, the seasonal moving average lag, s, the length of the cyclical pattern [13]. An ARIMA model is developed with four synergistic steps including time series stationary, model identification, parameter estimation and diagnostic checking [19].

Initially, the time series must be stationary. Log transformation, non-seasonal and seasonal differences are frequently used to stabilize the time series [14]. The Augmented Dickey-Fuller (ADF) test can determine whether the differenced time series is stationary or not [19].

Secondly, the Autocorrelation function (ACF) graph and partial autocorrelation (PACF) graph were employed to determine the possible values of p, d, P and D. Generally, we can choose more than one plausible models in this step.

Subsequently, we removed some unqualified models by the parametric and residual tests: the parametric test is statistical significance (p<0.05) and the residual test must show a white noise sequence using the Box-Jenkibs Q test.

Finally, the Akaike information criterion (AIC) and Schwarz Bayesian information criterion (SBC) were used to select the preferred model [22]. The model with the lowest AIC and SBC values was considered the best model. If the AIC and SBC values of these plausible models were nearly equal, the model with the higher R^2^ value was selected.

### Construction of the basic GRNN model

The GRNN model was primarily proposed and developed by Specht [24]. It is a universal approximator for smoothing factors based on non-linear regression theory. The GRNN consists of four layers: the input layer, pattern layer, summation layer and output layer [14]. The relationship between each pair of the input X and the observed output Y are examined by the network to deduce the inherent function [15]. The following equation summarizes the GRNN logic in an equivalent nonlinear regression formula:

$$E[Y/X]=(\int_{-\infty }^{\infty }Yf(X,Y)dY)/(\int_{-\infty }^{\infty }f(X,Y)dY)$$

Where X means the input vector (X_1_, X_2_,…, X_n_) which consists of n predictor variables, Y denotes the output values predicted by the GRNN. E[Y/X] is the expected value of the output Y given an input vector X, and f(X,Y) is the joint probability density of X and Y [25].

The structure of the basic GRNN model can be expressed as an (N-1) GRNN model, which means it is an N-dimensional input and one-dimensional output GRNN model. Moreover, the smoothing factor is the only parameter of the network [26]. Obviously, the two parameters (N and the smoothing factor) play an important role in constructing the basic GRNN. However, there are many possible values of these parameters. The best values of the parameters need to be determined in order to find the optimal GRNN model. Therefore, a basic GRNN model is constructed with four steps.

Initially, the original data are divided into two parts: the last two data sets as the testing set and the rest as the training set.

Subsequently, the training network was tested for a series of smoothing factors and N values to select the best smoothing factor and N values at which the RMSE of the network was the lowest.

Finally, the last N data of the original data were used as the input part to predict the future data via the best GRNN model.

### Development of the hybrid ARIMA-GRNN model

Extracting the linear information from the actual data is what the ARIMA model specializes in, but the residuals consist of non-linear information which the model cannot analyze. Fortunately, this information can be analyzed by the GRNN network. The hybrid ARIMA-GRNN model combined the advantages of the two basic model to mine the information of the data adequately. We used the fitting incidence of the ARIMA model as the input variable and the actual incidence as the manipulated value to develop the hybrid ARIMA-GRNN model. To determine the optimal smoothing factor, two samples were randomly selected as the testing data and the rest were employed to train the network [22]. The training network was tested for a series of smoothing factor to select the best smoothing factor at which the minimum RMSE of the network was the lowest. Subsequently, the forecasted values created by the ARIMA model were used as the enter values of the hybrid model, so then the combined model could output the predictive values [23].

### Comparison with the three models in simulation performance

The fitting and forecasting effect of these three models was estimated using the mean square error (MSE), root mean square error (RMSE) mean absolute percentage error (MAPE) and mean absolute error (MAE) [27, 28]. Eviews 8.0 was used to create the ARIMA model, the single GRNN model and hybrid ARIMA-GRNN model were constructed with Matlab2012b.

## Results

### Single ARIMA model

**Fig 1:**
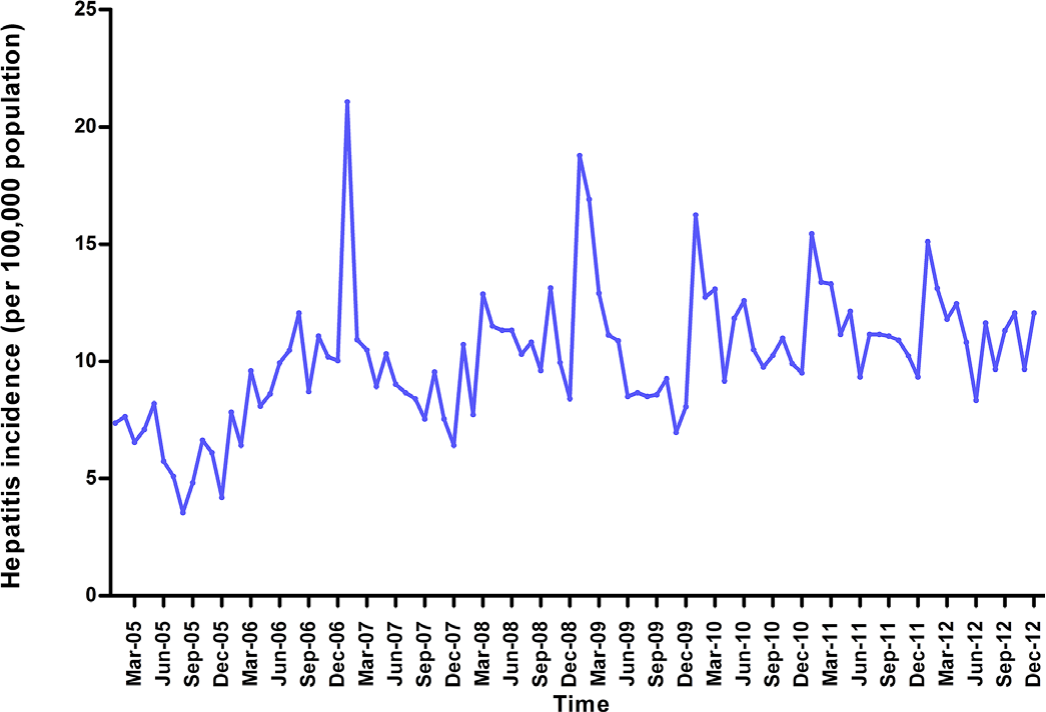
Monthly incidence of hepatitis from January 2005 to December 2012.

The monthly hepatitis incidence data from January 2005 to December 2012 in Heng County was used for model fitting (Fig 1). As can be seen in the Fig 1, the hepatitis incidence shows seasonal variation (s = 12) and a mildly rising trend, which showed the time series was not stationary. We made a log transformation, non-seasonal (d = 1) and seasonal difference (D = 1) to eliminate numerical instabilities, after these steps, the result of the ADF test (Table 1) was statistically significant (p<0.001), which showed that the time sequence was stationary.

**Table 1:** The ADF test of the transformed hepatitis incidence series.

		t-Statistic	p-value
Augmented Dickey-Fuller test statistic		-5.1601	0.0000
Test critical values	1% level statistic	-3.5065	0.01
	5% level statistic	-2.8947	0.05
	10% level statistic	-2.5845	0.1

**Fig 2:**
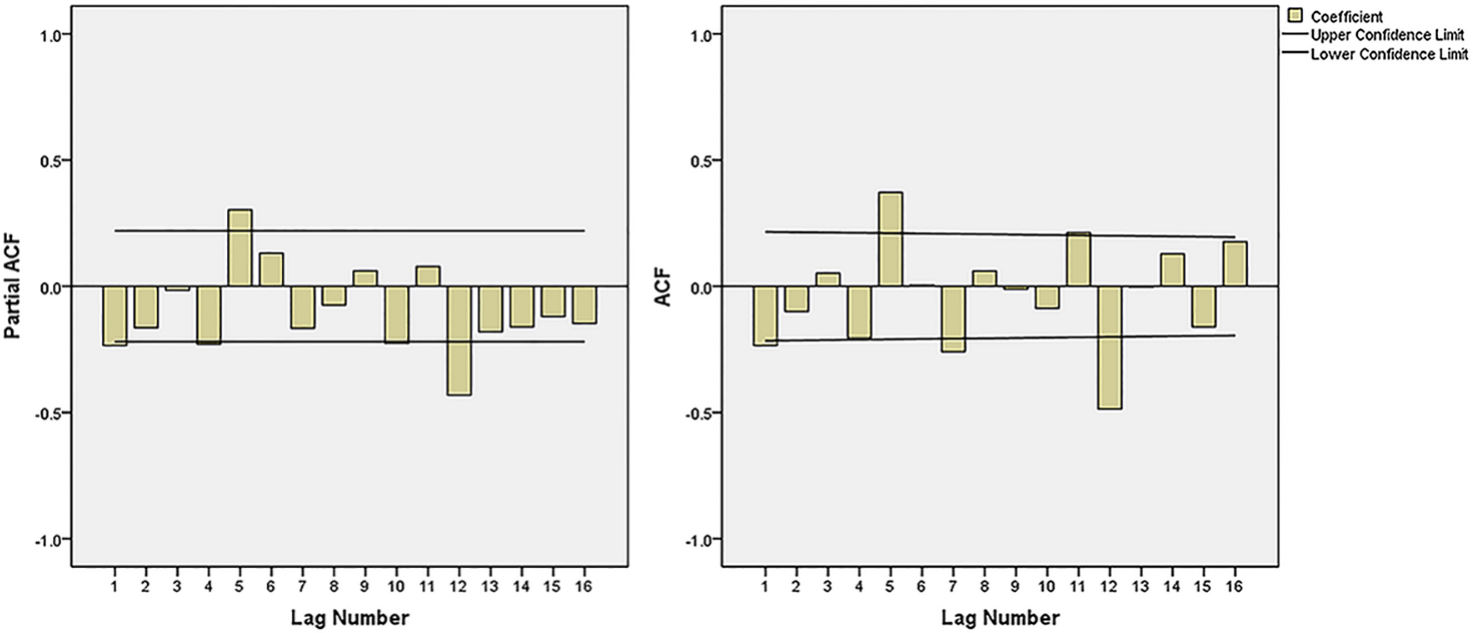
The ACF and PACF graphs of transformed hepatitis incidence series. ACF = the autocorrelation function graph and PACF = partial autocorrelation graph. The possible values of q and Q were 1, 2, 3 and 1 basic on the ACF graph, and the possible values of p and P were 1, 2, 3 and 1 basic on the PACF graph.

The ACF graph and PACF graph (Fig 2) were used to explore the parameters of the ARIMA model. By analyzing Fig 2, we choose several models, but some of them did not pass the model parameter or residual tests. Finally, three appropriate models were filtered: ARIMA (0,1,1)(1,1,1)_12,_ ARIMA (0,1,2)(1,1,1)_12_ and ARIMA (1,1,1)(1,1,1)_12_.The AIC and SBC values of the three models are shown in Table 2, where we can see that these three models had similar AIC and SBC values. Compared with the other models, the ARIMA (0,1,2)(1,1,1)_12_ model had the best R^2^ and AIC values, and thus was the most suitable model. Table 3 shows the parameters text results. The residual test of this model showed a white noise sequence (p>0.05).

**Table 2:** The parameters of the three ARIMA models.

Model	AIC	SBC	R^2^
ARIMA(0,1,2)(1,1,1)_12_	-1.0542	-0.9268	0.6056
ARIMA(0,1,1)(1,1,1)_12_	-1.0492	-0.9536	0.5922
ARIMA(1,1,1)(1,1,1)_12_	-1.0539	-0.9255	0.5859

ARIMA = the autoregressive integrated moving average; AIC = Akaike information criterion and SBC = Schwarz Bayesian information criterion; MAPE = mean absolute percentage error.

**Table 3:** Estimate parameters of the ARIMA (0,1,2)(1,1,1)_12_ model.

Variable	Coefficient	Std. Error	t-Statistic	p-value
SAR(12)	-0.3845	0.0984	-3.9069	0.0002
MA(1)	-0.5616	0.1033	-5.4364	0.0000
MA(2)	-0.3687	0.1040	-3.5442	0.0007
SMA(24)	-0.4784	0.1475	-3.2429	0.0018

ARIMA = the autoregressive integrated moving average; SAR(12) = Seasonal moving average, lag12; MA(1) = Moving average, lag1; MA(2) = Moving average, lag2; SMA(12) = Season Moving average, lag12.

### Basic GRNN model

**Fig 3:**
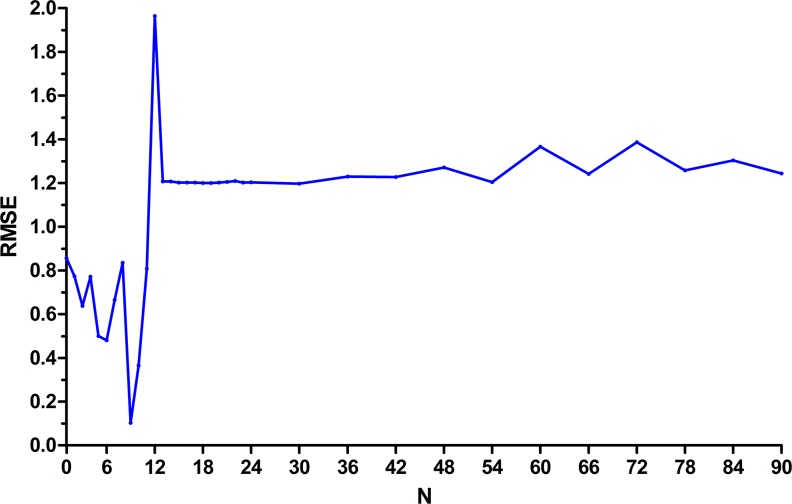
The RMSE of each basic GRNN models. RMSE = root mean square error; N = the number of input of the basic GRNN model. When the N was 9, the basic GRNN model had the minimum RMSE.

**Fig 4:**
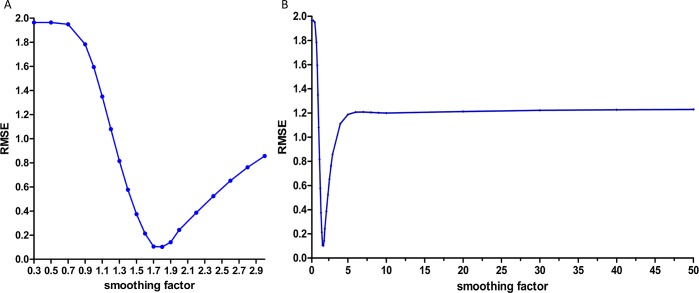
The selection of the basic GRNN model. GRNN = the generalized regression neural network. (A) The smoothing factor between 0.3 and 3.0 with an interval of 0.1 or 0.2 were selected to find the minimum RMSE for the basic GRNN model. The GRNN model has lowest RMSE when the smoothing factor came to 1.8. (B) The RMSE showed increase trend when the smoothing factor was higher than 0.3 or lower than 3.0.

The samples from January 2005 to December 2012 were selected to develop the network. We selected the morbidity of November 2012 and December 2012 as the testing samples and the rest of the data were used to train the network. Thus, N has the potential to take ninety different values, ninety basic GRNN models were developed to explore the best value of N. To determine the optimal smoothing factor for each network, we tested a series of smoothing factors to select the smoothing factor at which the minimum RMSE of the network was the lowest. Fig 3 shows the RMSE of these constructed networks. As can be seen in Fig 3, the basic GRNN model with nine-dimensional input and one-dimensional output had the minimum RMSE. So, we used the previous nine monthly incidences to predict the next one. The optimal smoothing factor of the best network was 1.8 (Fig 4).

### Hybrid ARIMA-GRNN model

**Fig 5:**
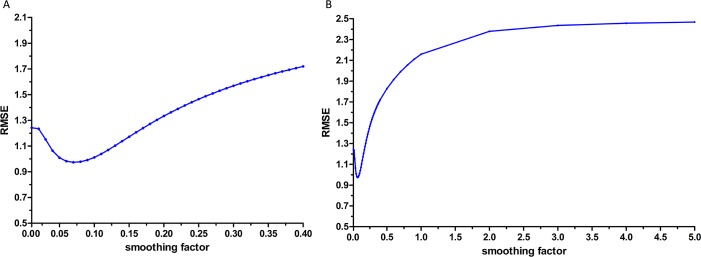
The selection of the ARIMA-GRNN model. ARIMA = the autoregressive integrated moving average; GRNN = the generalized regression neural network. (A) The smoothing factor between 0.01 and 0.40 with an interval of 0.01 were selected to find the minimum RMSE for the GRNN model. The GRNN model has lowest RMSE when the smoothing factor came to 0.07. (B) The RMSE showed increase trend when the smoothing factor was higher than 0.40 or lower than 0.01.

The morbidity data from February 2008 and December 2012 were randomly used as the testing samples for the GRNN model. When the smoothing factor was 0.07, the hybrid model had the lowest RMSE (Fig 5). Therefore, 0.07 was selected to as the most appropriate smoothing factor to develop the GRNN model. Subsequently, the forecasting outcomes of the ARIMA model from January 2013 to December 2013 were selected as the entry value of the GRNN model, and the output values were the predictive values of the combined ARIMA-GRNN model.

**Fig 6:**
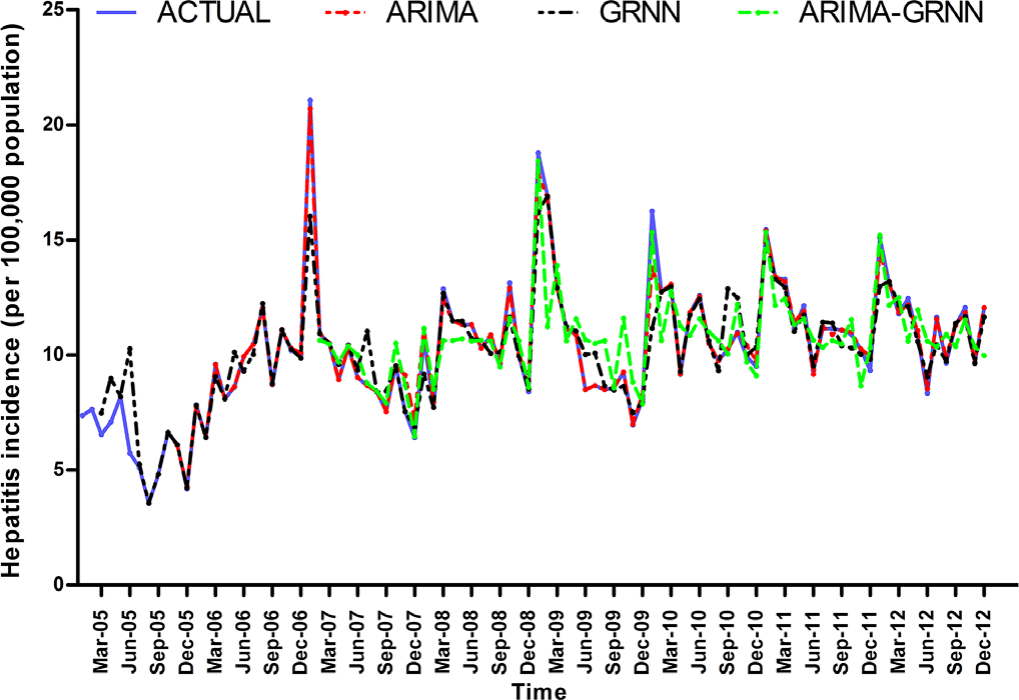
The fitting curves of the three models and the actual hepatitis incidence series. ARIMA = the autoregressive integrated moving average; GRNN = the generalized regression neural network.

**Fig 7:**
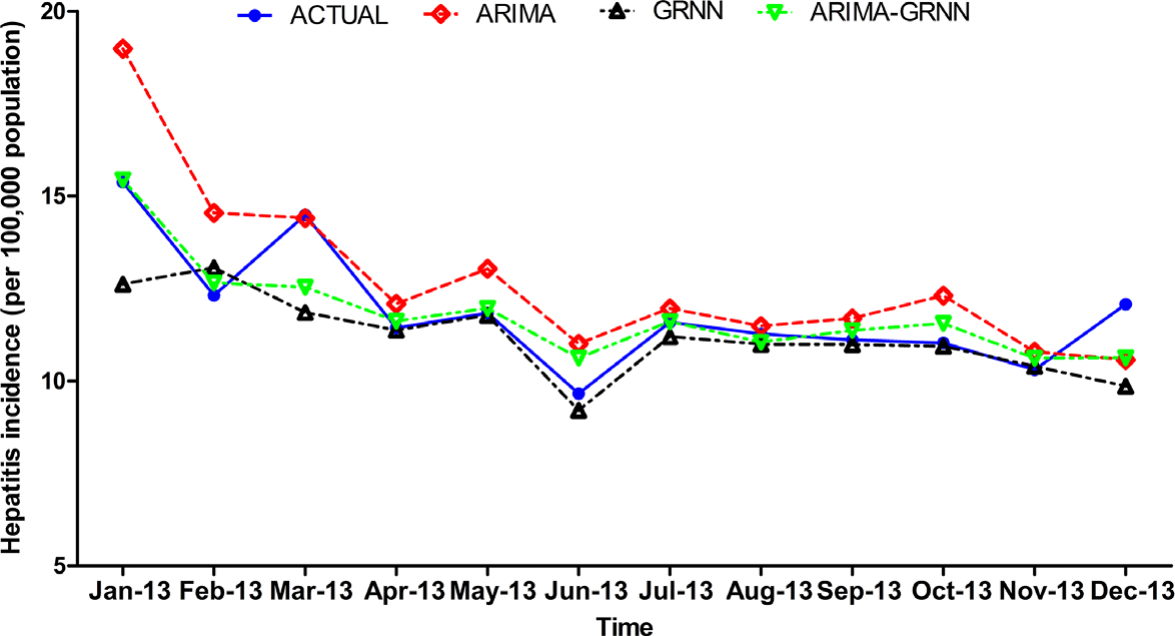
The forecasting curves of the three models and the actual hepatitis incidence series. ARIMA = the autoregressive integrated moving average; GRNN = the generalized regression neural network.

Finally, these three models were selected to forecast hepatitis morbidity in Heng County from January 2013 to December 2013. The fitting and prediction curves of the three models are depicted in Figs 6 and 7. The forecasting performance parameters of the three models for the fitting and validation parts are shown in Table 4.

**Table 4:** The fitting and forecasting performance of the three models.

	Fitting part				Validation part			
Prediction error	MAPE	MAE	MSE	RMSE	MAPE	MAE	MSE	RMSE
ARIMA	0.1115	1.2045	2.4215	1.5561	0.0925	0.9173	1.2322	1.1100
GRNN	0.0150	0.1595	0.2233	0.4726	0.0625	0.8266	1.7090	1.3073
ARIMA-GRNN	0.0878	0.8820	0.8820	0.9391	0.0445	0.1933	0.2176	0.4665

ARIMA = the autoregressive integrated moving average; GRNN = the generalized regression neural network; MAPE = mean absolute percentage error; MAE = mean absolute error; MSE = the mean square error; RMSE = root mean square error.

## Discussion

Although the traditional ARIMA model and the basic GRNN model did well in hepatitis incidence forecasting, the hybrid model showed better performance in terms of data prediction. Interestingly, the basic GRNN model was superior in data fitting among three models. It is worth noting that the model was used to predict hepatitis incidence, so the forecasting performance should assessed first. Moreover, the hybrid model also did well in term of data fitting, so we can entirely exclude the possibility that the high performance of the combined model in forecasting was caused by accidental factors. Hence, in this study, we believe that the hybrid ARIMA-GRNN model is a decision-making tool with enormous potential for making the correct public health policy decisions and mobilizing much needed resources.

The traditional ARIMA model was used as the baseline model for evaluating the performance of the combined model in previous researches [21, 23, 29]. However, it is possible that the basic GRNN model may be better than the hybrid one. So we developed three forecasting models to predict the monthly incidence of hepatitis. We came to the same conclusion that the hybrid model outperformed the ARIMA model [17, 19, 25]. Furthermore, we also compared the performance parameters of the hybrid model and the basic GRNN model; the hybrid model was also superior for data forecasting. Meanwhile, using three models, we further tested three major infectious diseases in China, tuberculosis, hemorrhagic fever and syphilis. The incidence data (2004–2012) came from the public health science data center of Chinese Center for Disease Control and Prevention (Chinese CDC) (website: http://www.phsciencedata.cn/Share/ky_sjml.jsp). The results (S2–S4 Tables) also support our conclusion. Thus, the combined ARIMA-GRNN model was identified as the best forecasting model. Moreover, we used it to predict the incidence of hepatitis in the next 12 months, and the prediction accuracy remained high.

The basic GRNN model was developed as a new potential tool for infectious diseases incidence prediction field in recent years [30]. Han, et al [20] constructed this network with one-dimensional input and one-dimensional output to forecast the incidence of blood and sexually transmitted diseases. It is noteworthy that these authors didn’t test the other input and output construction of GRNN models. They could not absolutely make the conclusion that this model was the best. In this study, we presented a better method to develop the optimum GRNN model. We developed several basic GRNN models to find the best input and output construction of the model, in which the error of the model was the lowest. As can be seen in Fig 3, when the N was between 1 and 12, the error of the network obviously fluctuated. Conversely, the error was higher and showed a stable trend when N was higher than 12. This may reduce our workload when we update the GRNN model for hepatitis incidence in Heng County, as we just need to develop 12 networks of different construction for the model to be sufficient.

Seasonal variation was found in the time series, as the reported incidence hepatitis was highest during the spring but lowest in the winter. This conclusion was also made in other studies on the seasonality of hepatitis in different regions of China [31–33]. The annual Spring Festival, the most important Chinese traditional festival, can be used to explain the seasonal trend in Heng County. During the Spring Festival, there are enormous population movements throughout China and a large number of families or friends get together for the holiday [34, 35]. Thus, we suggest that the peak time of hepatitis incidence, especially the morbidity of hepatitis A and E which are transmitted by the fecal-oral route, may be partly attributed to huge dinner parties [36–39]. Furthermore, Heng County is famous for eating fresh fish, which is a potential high-risk behavior that may cause inflammatory infection of the liver [40–42]. Therefore, some measures should be taken to prevent the hepatitis transmission during the Spring Festival.

With the help of the hybrid model, it is reasonable for the government to allocate health resources to control the epidemic efficiently. If prediction results continue to rise, the government should be prepared to allocate more resources into health interventions in advance. It also shows that the currently used intervention strategies may be inadequate. Moreover, it can be used to assess the protective effect of the hepatitis vaccine. After vaccination, the model may show that the vaccine is effective if the actual incidence is lower than the predicted result. Above all, the hybrid model will play an important role in controlling the hepatitis epidemic in Heng County. It can also be extended to other regions of Guangxi.

Although the hybrid ARIMA-GRNN model showed satisfactory forecasting performance, several limitations of this model should be noted. Initially, the hybrid model was merely used for short-term prediction [43]. Hence, the model should be constantly updated in order to maintain prediction performance. Subsequently, the hepatitis epidemic is influenced by many elements, such as environmental changes, human behaviors, health interventions and so on. However, the model only considers the time factor. A single factor model is not compatible with complex epidemic problems, which are inherently noisy. Therefore, the multi-factor model has better prospects [44–47].

## Conclusions

In general, the combined ARIMA-GRNN model was the best prediction model, and is a potential decision- supportive tool for the Department of Disease Control and Prevention of Heng County to control the hepatitis epidemic.

## Supporting Information

S1 Table The data of hepatitis morbidity in Heng County from January 2005 to December 2013. (XLSX)

S2 Table The fitting and forecasting performance of three models for the tuberculosis incidence in China from 2004 to 2012. (DOCX)

S3 Table The fitting and forecasting performance of three models for the hemorrhagic fever incidence in China from 2004 to 2012. (DOCX)

S4 Table The fitting and forecasting performance of three models for the syphilis incidence in China from 2004 to 2012. (DOCX)
