# Supplementary material for: Application of a Combined Model with Autoregressive Integrated Moving Average (ARIMA) and Generalized Regression Neural Network (GRNN) in Forecasting Hepatitis Incidence in Heng County, China
Source: PLoS One. 2016 Jun 3;11(6):e0156768. doi: 10.1371/journal.pone.0156768 (PMC4892637; doi:10.1371/journal.pone.0156768)
Supplement: S4 Table — (DOCX) [file pone.0156768.s004.docx]

**Supplimentary Table 4. The fitting and forecasting performance of three models for the syphilis incidence in China from 2004 to 2012.**

|  | **Fitting part**  **(Prediction errors)** | | | **Validation part**  **(Prediction errors)** | | |
| --- | --- | --- | --- | --- | --- | --- |
| **Models** | **MAE** | **MAPE** | **MSE** | **MAE** | **MAPE** | **MSE** |
| ARIMA | 1.9608 | 0.0436 | 0.0249 | 0.2642 | 0.1064 | 0.0745 |
| GRNN | 0.1659 | 0.0853 | 0.0467 | 0.1964 | 0.0776 | 0.0525 |
| ARIMA-GRNN | 0.0388 | 0.0220 | 0.0027 | 0.1669 | 0.0695 | 0.0400 |

The data incidence came from the public health science data center of Chinese Center for Disease Control and Prevention (website: <http://www.phsciencedata.cn/Share/ky_sjml.jsp>). ARIMA=the autoregressive integrated moving average; GRNN=the generalized regression neural network; MAPE=mean absolute percentage error; MAE= mean absolute error; MSE=the mean square error.
